# Recent Advances of Adipose-Tissue-Derived Mesenchymal Stem Cell-Based Therapy for Retinal Diseases

**DOI:** 10.3390/jcm12227015

**Published:** 2023-11-09

**Authors:** Lucia Finocchio, Marco Zeppieri, Andrea Gabai, Leopoldo Spadea, Carlo Salati

**Affiliations:** 1 Department of Ophthalmology, University Hospital of Udine, 33100 Udine, Italy; luciafinocchio@gmail.com (L.F.);; 2 Eye Clinic, Policlinico Umberto I, La Sapienza University of Rome, 00142 Rome, Italy

**Keywords:** adipose tissue-derived mesenchymal stem cell, cell therapy, regenerative, retinal diseases

## Abstract

With the rapid development of stem cell research in modern times, stem cell-based therapy has opened a new era of tissue regeneration, becoming one of the most promising strategies for currently untreatable retinal diseases. Among the various sources of stem cells, adipose tissue-derived mesenchymal stem cells (ADSCs) have emerged as a promising therapeutic modality due to their characteristics and multiple functions, which include immunoregulation, anti-apoptosis of neurons, cytokine and growth factor secretion, and antioxidative activities. Studies have shown that ADSCs can facilitate the replacement of dying cells, promote tissue remodeling and regeneration, and support the survival and growth of retinal cells. Recent studies in this field have provided numerous experiments using different preclinical models. The aim of our review is to provide an overview of the therapeutic strategies, modern-day clinical trials, experimental models, and potential clinical use of this fascinating class of cells in addressing retinal disorders and diseases.

## 1. Introduction

The retina is a highly specialized neural tissue and represents the photosensitive part of the central nervous system that captures a light signal via the photoreceptors and converts it into electrical impulses that are transmitted to the visual cortex of the brain via the optic nerve. It is a complex multi-layered structure composed of several layers of morphologically and functionally different cell types that are interconnected by synapses and externally lined by a single outer layer of pigmented epithelial cells (retinal pigmented epithelium, RPE). Each layer can be subject to damage from insults, resulting in a loss of vision. Retinal diseases can be broadly classified into degenerative, inflammatory, vascular, and hereditary retinal disorders. Although retinal diseases have various causes and different etiologies, a common characteristic is the death or dying of the specialized retinal cells and a loss of integrity in the retina structure or the degeneration of the photoreceptors (PRs), ultimately resulting in visual impairment. The adult mammalian retina has inadequate regenerative capacity and, thus, PRs or RPE cell death can lead to irreversible vision loss. In particular, age-related macular degeneration (AMD), diabetic retinopathy (DR), and hereditary retinal diseases are leading causes of irreversible vision loss and causes of significant suffering, as well as a high socio-economic burden [1].

To date, the treatment options for retinal diseases have been very limited and primarily consist of laser photocoagulation for DR, microsurgical interventions, and intravitreal administration of vascular endothelial growth factor inhibitors (anti-VEGF) or other drugs that slow the progression of the disease process but do not arrest it or promote recovery. The loss of specialized retinal cells and local inflammatory reactions are the main contributing factors for the progression of retinal degenerative diseases and the inhibition of inflammation; support for the surviving retinal cells appears to be prospective in approaches to manage these diseases. Researchers are, therefore, constantly searching for ways to reduce the incidence of blindness by preventing disease development and progression or by finding ways to repair damaged retinal tissues.

Advances in stem cell research have allowed for the generation of new cells and tissues that have been proposed for transplantation to benefit patients with blinding disorders. Stem-cell-based therapy is becoming one of the most promising strategies for currently untreatable retinal diseases. For example, a recent study suggested the paracrine properties of various types of stem cells could contribute to the inhibition of inflammatory processes that would otherwise threaten the survival of retinal cells in certain pathological diseases [2].

Adipose-derived stem cells (ADSCs) are a particular class of mesenchymal stem cell that hold great promise for treating retinal diseases, both acquired and inherited, and have the potential to revolutionize the concept of retinal therapeutics [3]. The purpose of this review is to provide an overview of the therapeutic strategies, modern-day clinical trials, experimental models, and potential clinical use of this class of cells in addressing retinal disorders and diseases and to highlight the current achievements and limitations of this promising technology.

## 2. Stem Cells in Ophthalmology

Stem cells (SCs) are unspecialized cells of the human body that are present in the embryonic, fetal, and adult stages of life, and they play a crucial role in embryonic development, tissue repair, and regeneration. This is achieved thanks to their ability to both replicate and proliferate extensively (self-renewal), thereby maintaining a constant pool of undifferentiated cells and differentiating into multiple specialized cell types (potency), forming various tissues and organs [4]. SCs can be classified into totipotent, pluripotent, and multipotent. Totipotent SCs (zygote) have the highest differentiation potential and are able to divide and differentiate into any cell of the organism: totipotent SCs are responsible for forming the embryo and all extra-embryonic structures. Pluripotent SCs (PSCs) derive from the totipotent SCs and form cells of all three germ layers but not extraembryonic structures. Examples of PSCs are represented by embryonic stem cells (ESCs), derived from the inner cell mass of preimplantation embryos, and induced pluripotent stem cells (iPSCs), which arise from the epiblast layer of implanted embryos.

Multipotent stem cells have a narrower spectrum of differentiation than PSCs and generate distinct and specialized cells of one specific germ layer. Examples of multipotent SCs are mesenchymal stem cells (MSCs) and hematopoietic stem cells. Finally, oligopotent SCs, like myeloid stem cells, can differentiate into several cell types, while unipotent SCs have the narrowest differentiation capabilities and the special ability to divide repeatedly but are only able to form one cell type [5,6,7]. In view of their differentiating potential, SCs can potentially be used to repair or replace damaged tissues, in most cases avoiding the risks of rejection typically associated with traditional transplant methods [8].

In ophthalmology, SC research is rapidly advancing and has shown great promise in treating a variety of eye conditions. The human eye combines tissues from neuroectodermal (e.g., retina), ectodermal (e.g., lens and cornea), and mesodermal lines and is an optimal target for stem cell transplantation therapies due to the high number of people affected by eye disease and the relative ease of accessibility to ocular tissues [7]. The use of SCs has been advocated to restore vision in patients with many eye diseases, and SCs have not gone unnoticed in the context of retinal diseases, with interest in several pathologies, including acquired retinal diseases (e.g., age-related macular degeneration (AMD)) and inherited retinal pathologies [9,10].

Researchers are exploring the use of ESCs, iPSCs, and adult stem cells to generate RPE cells or photoreceptor cells for transplantation. In particular, iPSCs represent a unique in vitro model, allowing for the generation of retinal progenitor cells [4], and human pluripotent stem cells (hPSCs) have shown potential in the replacement of the retinal pigment epithelial cell (RPE) and photoreceptors in the context of AMD. Adult SCs have also been evaluated in various clinical trials in the field of ophthalmology. Among them, neural stem cells (NSCs), bone marrow stem cells (BMSCs), and mesenchymal stem cells (MSCs) have been assessed. NSCs can be isolated from the developing or mature mammalian central nervous system (CNS) and have been shown to secrete trophic factors with the potential for photoreceptor neuroprotection [11].

BMSCs may be useful to prevent graft vs. host disease in corneal transplantation and have also been studied in retinal diseases, showing a paracrine trophic effect on the degenerating ischemic retina [12]. Finally, MSCs are the most commonly studied multipotent SCs in ophthalmopathies and have been found in various fetal tissues, in extraembryonic tissues, like the placenta, the umbilical cord, and the amniotic fluid, and in adult tissues (bone marrow, peripheral blood, adipose tissue, dermis, synovium, periosteum, cartilage, skeletal muscle, fallopian tube, menstrual blood, gingiva, dental tissue, and eye).

Umbilical cord MSCs have shown anti-inflammatory and immune-privilege properties, and they can differentiate into corneal epithelial, stromal, and endothelial cells [13], but it is placental and adipose-derived MSCs that have been evaluated most in clinical trials. In particular, human adipose-derived stem cells (hADSCs) resemble BMSCs in terms of morphology, proliferation, and multipotency and have been shown to have neuroprotective and neurogenerative properties in the treatment of retinal degeneration [14] and corneal disease [15]. Dental pulp stem cells (DPSCs) are another category of SCs that have been studied for corneal repair [16] and for the treatment of glaucoma and retinal pathologies [17].

An interesting type of adult SC capable of producing both CNS and mesoderm-associated lineages is represented by RPE stem cells (RPESCs), which proliferate pathologically in specific conditions and result in retinal disease [18]. In the field of corneal regeneration, SCs derived from various sources, such as limbal stem cells (LSCs) or iPSCs, can differentiate into corneal epithelial cells, and can be used to regenerate and repair damaged corneal tissue and help restore vision [19]. Bone marrow MSCs (BM-MSCs) have been shown to differentiate into corneal epithelial-like cells in vivo in rat-damaged corneas [20], and Calonge et al. successfully used them combined with an amniotic membrane transplant in the treatment of LSC deficiency [21].

In terms of optic nerve regeneration, research is underway to develop methods to differentiate SCs into retinal ganglion cells (RGCs), which make up the optic nerve, and integrate them into the existing neural circuitry [22]. iPSCs/ESCs represent the first source studied for optical nerve regeneration: hESCs have shown the ability to propagate indefinitely and to differentiate into many different types of cell lineages, including retinal ganglion cells (RGCs) [4]. The presence of MSCs in the trabecular meshwork (TM), which is an avascular tissue that is located within the uvea that controls the humor aqueous’ outflow, has been suspected for years and was confirmed and propagated in vitro by Ding et al. [23].

The aforementioned findings are only some examples of the key roles of stem cell therapy in ophthalmology. While this field holds immense potential, it is essential to conduct further research, and clinical trials are essential to ensure the safety and efficacy of this relatively new technology. Additionally, regulatory approval and ethical considerations are critical aspects that need to be addressed before the widespread adoption of stem-cell-based treatments in ophthalmology.

## 3. Adipose-Derived Mesenchymal Stem Cells (ADSCs)

Adipose tissue is present in all mammalians and some non-mammalian species, where it mainly serves as an energy reservoir in lipidic form, exerting this endocrine function throughout the body, in subcutaneous tissues, as visceral fat in the intraperitoneal compartment, and padding many vital structures. Like the dermis, bone, cartilage, and blood vessels, fat tissue develops from the embryonic mesoderm. Mature adipocytes are produced by a process known as adipogenesis, where precursor cells called preadipocytes proliferate and differentiate under the control of complex hormonal, neuronal, and paracrine pathways.

MSCs studied for regenerative applications derive from white adipose tissue and can be easily collected via liposuction. This minimally invasive procedure can provide a high number of multipotent SCs, which makes it particularly advantageous when compared to scavenging from other sources, such as bone marrow and dental pulp [24,25]. In particular, ADSCs are multipotent cells naturally residing in the adipose stroma that can be isolated in large quantities through enzymatic digestion and centrifugation of the lipoaspirate material, and they can be subsequently separated and cultured. ADSCs present fibroblastic morphology and, differently from other lipoaspirate cells, bear the potential to differentiate in all three mesodermal lineages (osteogenic, adipogenic, and chondrogenic). This multilineage potential is confirmed by the histological characteristics, specific cell surface markers, and the expression of several multilineage-specific genes. Moreover, ADSCs show a high proliferative capacity, and their differentiation into multiple cell lineages can be induced by referring to specific culture conditions and signaling factors [26].

**Figure 1:**
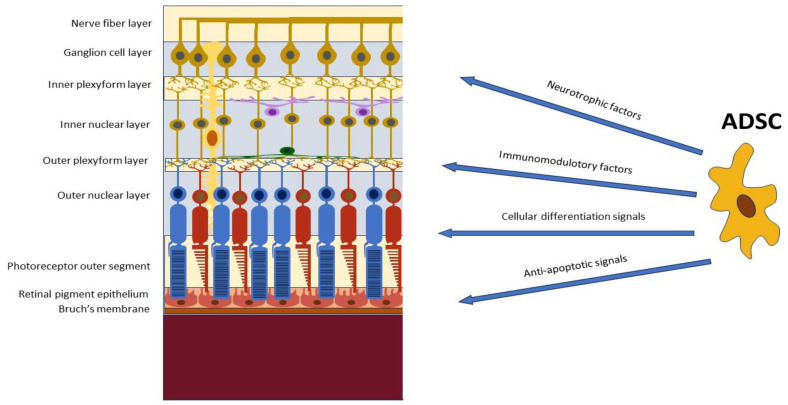
Potential applications of adipose-derived stem cells (ADSCs) in the treatment of retinal pathologies.

ADSCs have also been found to bear anti-inflammatory effects by secreting immunomodulatory cytokines and growth factors [27] and to present migratory and homing abilities targeting their action in tissue remodeling and regeneration [28,29,30]. Interestingly, ADSCs express low levels of oxygen reactive species (ROS) and high levels of glutathione, conferring in them significant resistance to oxidative stress. This represents a considerable advantage for potential applications of ADSCs in the treatment of various forms of retinal degeneration where oxidation plays a relevant role [31] (Figure 1).

Stem cell-based treatments have been demonstrated to be safe and effective in several preclinical studies, both in vitro and in vivo. Nevertheless, considerable issues remain to be resolved to apply them in clinical settings. Firstly, there is not yet an agreement on their preparation due to the heterogeneity in cell-sampling procedures and culture methods of published studies. Further, the survival capacity of SCs in vivo is still controversial, with reports of long-term survival of MSCs in immunocompetent individuals opposed to evidence of the short life of in vitro cultured stem cells after their administration [32]. To this extent, ocular stem cell therapies have some advantages, with the eye being an immunologically privileged organ. Nevertheless, the interaction between the SCs’ immunomodulatory action and the proinflammatory events occurring in the diseased eye could trigger the proliferation of aggressive proinflammatory cells [33].

The administration route is also a crucial factor, especially for the treatment of ocular disease. Recent observations proved that only a small portion of mesenchymal stem cells reach the eye when they are injected intravenously, making intraocular delivery methods preferable when studying therapeutic effects in the ocular tissues [33]. However, even after the intravitreal injection of cells in animal models of retinitis pigmentosa, only a few of them were detectable in the vitreous cavity, with significant side effects [34]. Therefore, other techniques to deliver mesenchymal cells intraocularly were explored, such as subtenon and suprachoroidal injection, for the same disease [35,36]. More recently, their ability to release neuroprotective, antiapoptotic, and pro-proliferative cytokines was demonstrated in vivo, after the subretinal implantation of cells, in mice models of retinal degeneration and in vitro on ischemic RGCs [37,38]. Exosomes derived from ADSCs and delivering micRNA-222 to the retinal cells of rabbits with induced diabetic retinopathy showed the ability to repair retinal damage; the increased expression of micRNA-222 was associated with regenerative changes in the retina. This therapeutic effect was seen via both the systemic and ocular (intraocular and subconjunctival) administration of rabbit adipose tissue-derived MSCs exosomes that can be considered novel therapeutic vectors [39].

## 4. Methods

We conducted a search of the literature published between 1 January 2002 and 30 June 2023 using MEDLINE (PubMed). The database was first searched using the keywords “stem cells, adipose-derived mesenchymal stem cells, AND retina, AND retinal diseases”. We considered only studies in English and those with an abstract. The reference lists of all retrieved articles were assessed to identify additional relevant studies. The research of articles was performed using PubMed (https://pubmed.ncbi.nlm.nih.gov, accessed on 1 June 2023).

Only articles with an abstract were considered. Studies in which small case series were described and those that assessed surgical techniques were analyzed. Each study was independently assessed by at least two reviewers (L.F, A.G, and M.Z), and rating decisions were based on the consensus of the reviewing authors. A total of 130 references were included in this review. This search strategy was limited considering the vast literature, which could have, thus, potentially and unintentionally excluded opinion leaders in this field of research.

## 5. Adipose-Derived Stem Cells (ADSCs) in Retinal Diseases

ADSCs have demonstrated various beneficial effects in the treatment of retinal diseases, including the modulation of retinal inflammation and immune responses, promotion of retinal cell survival and regeneration, and enhancement of retinal vascularization and neuroprotection. ADSCs possess the capacity to secrete a range of bioactive molecules, including growth factors, cytokines, and extracellular vesicles, which play a crucial role in the therapeutic effects observed in retinal disease. Known paracrine factors secreted by ADSCs include matrix metalloproteinase inhibitors, vascular endothelial growth factor (VEGF), [40] platelet-derived growth factor (PDGF), tumor necrosis factor-alpha (TNF-alpha), [41] human growth factor, various cytokines, hepatocyte growth factor (HGF) [42], transforming growth factor beta-1 (TGF beta-1) [43], etc. These paracrine factors can exert anti-inflammatory, anti-angiogenic, and neuroprotective effects, creating a conducive microenvironment for retinal tissue repair and regeneration [44].

In addition, ADSCs have been shown to differentiate into various retinal cell types, such as RPE cells and PRs, with the potential to replace cells damaged or lost in a specific degenerative condition. This ability to differentiate into functional retinal cells may, therefore, contribute to the restoration of retinal structure and function [45]. In particular, Huang et al. reported the in vitro differentiation of human ADSCs into retinal progenitors, RGCs, and PR cells expressing characteristic retinal cell markers when treated with noggin-related protein-1, IGF-1, and they exhibited a glutamate-evoked calcium response [46]. Amirpour et al. reported that the neurotrophic factors present in an hADSC-conditioned medium could induce hADSCs into eye-field neuroectoderm (EFN) cells [47].

Moreover, human ADSCs cultured with a conditioned medium from RPE showed the ability to differentiate into cells expressing typical RPE markers RPE65, cytokeratin 8, and bestrophin and acquired high proliferative and migratory ability in vitro [48]. It has been shown that ADSCs transduced with human transcription factor Paired box 6 protein (PAX6, 5a) and cultured in media supplemented with fibronectin have also been shown to differentiate into retinal progenitors, PRs, and RPE cells expressing cone-rod homeobox protein (CRX), rhodopsin, and RPE65 [49].

### 5.1. Modulation of Retinal Inflammation and Immune Responses

Retinal diseases often involve chronic inflammation and immune dysregulation, leading to progressive tissue damage. The immunomodulatory properties of ADSCs may attenuate inflammation and modulate immune responses [50,51,52,53]. Several research groups have demonstrated that ADSCs regulate the immune system through two primary mechanisms, directly via cell-to-cell communication and indirectly through the secretion of soluble mediators, growth factors, and extravascular vesicles [54,55,56]. In particular, ADSCs have been shown to exert immunomodulatory effects both in vitro and in vivo [57,58,59] by releasing various immunomodulatory proteins, including indoleamine 2, 3-dioxygenase 1 (IDO) and prostaglandin E2 (PGE2) [60,61]. ADSCs can also inhibit the activation and proliferation of immune cells, such as T cells and macrophages, and suppress the secretion of pro-inflammatory cytokines. Finally, they can also promote the generation of regulatory T cells and anti-inflammatory cytokines, creating an immune-regulatory environment that supports tissue healing and regeneration [62].

ADSCs are also able to act on vascular inflammatory responses and endothelial dysfunction.

An excess of reactive oxygen species (ROS) is involved in the process of apoptosis, whilst low cell levels of ROS in the cells activate receptor types and signaling pathways that influence proliferation. It has been demonstrated that the functional properties of ADSCs are under redox control [63], and although ADSCs are responsive to several stimuli, the primary factor is oxygen tension, and low oxygen levels (2–8% O_2_) are a key aspect of the cell niche. Absolute oxygen tension within adipose tissue is very low, and ADSCs exist in low-oxygen conditions in the body; [64] it is now clear that ROS generation associated with hypoxia increases the proliferation and survival of human ADSCs [65], and when a local injury occurs, ROS and endogenous factors such as chemokines are produced from damaged cells, inducing ADSC migration. This highlights the multiple roles of ADSCs in the protection of cells by modulating inflammation and immunity [66].

### 5.2. Promotion of Retinal Cell Survival and Regeneration

ADSCs have been shown to enhance retinal cell survival and regeneration in various retinal disease models. The ADSC-secreted paracrine factors can promote the survival of retinal cells, including PRs and RGCs, commonly affected in degenerative conditions. Furthermore, ADSCs can stimulate endogenous retinal progenitor cells, promoting their proliferation and differentiation into functional retinal cells. This regenerative potential of ADSCs contributes to the replacement of damaged cells and the restoration of retinal structure and function.

The results obtained in a large number of experimental and clinical studies demonstrated that either systemic or local (intracerebral or intraocular) injection of ADSCs had beneficial effects in the treatment of neural and retinal diseases. ADSCs engraft in injured tissues and produce neurotrophins, angio-modulatory, and immunoregulatory factors that suppress the detrimental immune response and promote the regeneration of injured neural and retinal cells [67,68,69,70].

An interesting and promising approach is the use of adipose tissue-MSC-derived exosomes (AT-MSC-Exos), nano-sized extracellular vesicles that contain all neurotrophins, immunoregulatory, and angio-modulatory factors secreted by their parental AT-MSCs. As cell-free products, AT-MSC-Exos address all safety concerns related to the transplantation of AT-MSCs. Accordingly, the injection of AT-MSC Exos has been considered an alternative therapeutic approach to AT-MSC-based therapy in the treatment of neural and retinal diseases [71,72,73,74,75].

### 5.3. Modulation of Retinal Vascularization and Neuroprotection

In retinal vascular diseases, such as DR, ADSCs have demonstrated the ability to enhance retinal vascularization by secreting pro-angiogenic factors that stimulate the formation of new blood vessels, thus improving retinal blood flow. It has been found that ADSCs differentiate into pericytes that can stabilize retinal vessels in multiple pre-clinical models of retinal vasculopathy, suggesting they may be useful as a protective and regenerative cellular therapy for retinal vascular disease [76,77]. In particular, when injected intravitreally, ADSCs enhance retinal microvascular stabilization in pre-clinical murine models of retinopathic vasculopathy [78].

ADSCs also secrete neurotrophic factors, such as brain-derived neurotrophic factor (BDNF) and hepatocyte growth factor, exerting neuroprotective effects by reducing oxidative stress, inhibiting apoptosis, and promoting the survival of retinal neurons [68].

It has been demonstrated in various animal models that ADSCs represent a potential tool in order to prevent diabetic retinopathy and provide an effective cytoprotective microenvironment in the retina of diabetic mice [39,76,77,79]. The studies performed by Cronk et al. conclusively established the feasibility of using ADSCs for stabilizing the retinal microvasculature in the DR model, supporting the utility of an allogeneic injection of ADSCs versus autologous or conditioned media approaches in the treatment of DR [80].

ADSCs are also a source of antioxidants, free radical scavengers, and heat shock proteins at the site of tissue insult [81]. These neuroprotective properties enhance the resilience of retinal cells against pathological insults and promote their functional recovery.

## 6. In Vitro Studies

In vitro studies explore ADSCs’ potential therapeutic applications. They can involve cell culture models, differentiation assays, and molecular analysis. In cell culture models, ADSC cultures are primarily used to evaluate their potential to differentiate into specific cell types and their overall behavior. Protocols that induce ADSCs to differentiate into RPE cells, PR-like cells, and other retinal cell types have been developed, allowing researchers to quantify the differentiation efficiency, integration potential, and functional properties of ADSC-derived retinal cells (Table 1).

ADSCs are known to play a role in retinal and photoreceptor cell proliferation [82]. Human ADSCs (hADSCs) have shown a trilineage potential to proliferate, migrate, and differentiate into RPE cells when exposed to an RPE-cell-conditioned medium [48]. Moreover, paired box 6 protein (5a) (PAX6 (5a)), a highly conserved transcription factor expression that has an essential role in the development of the vertebrate visual system, paired with a fibronectin-supplemented medium, is able to induce the differentiation of hADSCs into retinal progenitors, RPE cells, and PRs [49]. Xu et al. also found that orbital ADSCs isolated via direct explant culture showed earlier and stronger expression of markers, indicating retinal PR differentiation compared with those generated using the conventional enzyme method [83].

In differentiation assays, immunocytochemistry, gene expression analysis, and functional assays can be used to check the expression of retina-specific markers or the acquisition of functional properties of ADSCs, thus establishing the likeliness these cells have of integrating into the retina and replacing damaged tissue. Mannino et al. showed that the presence of growth factors produced by an RPE cell line (ARPE-19 cells) in tissue culture induces ADSCs to express neural differentiation markers typical of the neuronal and glial cells of the retina [84]. Studies have also shown that adding specific growth factors, like retinoic acid, fibroblast growth factor (FGF), or Wnt signaling activators, can promote the differentiation of ADSCs into RPE-like cells, which exhibit characteristic RPE morphology, express RPE-specific markers (e.g., RPE65, Bestrophin-1), and display functional RPE properties (phagocytosis of photoreceptor outer segments; production of RPE-associated factors) [45]. Similarly, by mimicking the developmental cues that guide PR differentiation, ADSCs have been induced to express photoreceptor-specific markers, such as recoverin, rhodopsin, and cone opsin. Molecular analysis is another useful tool as it evaluates the molecular characteristics of ADSCs and of their differentiated progeny, thereby identifying the signaling pathways, transcription factors, and regulatory molecules involved in ADSC-derived retinal cell functions.

### 6.1. Evaluation of Trophic and Paracrine Effects on Retinal Cells

As mentioned previously, ADSCs have been shown to promote a supportive microenvironment for retinal cell survival and function by means of paracrine abilities and the secretion of growth factors, cytokines, and chemokines. ADSCs can secrete a wide variety of neurotrophic factors (NTFs), such as hepatocyte growth factor (HGF), ciliary neurotrophic factor (CNTF), insulin-like growth factor (IGF) [89], basic fibroblast growth factor (FGF2), epidermal growth factor (EGF) [90], vascular endothelial growth factor (VEGF), nerve growth factor (NGF), brain-derived growth factor (BDNF), glial cell-derived neurotrophic factor (GDNF), neurotrophin-3 (NT-3), and platelet-derived growth factor (PDGF) [91]. These factors have a role in the survival, proliferation, and differentiation of retinal cells [92].

Dov et al. demonstrated that the transplantation of ADSCs significantly improved the recovery of retinal RGCs in organotypic ischemic retinas, probably via paracrine pathways, and advocated the potential of using these cells in future studies of regenerative therapy for ischemic retinas and ischemic RGCs [37]. Conditioned media from human ADSCs have also been shown to protect RPE and PR cells from oxidative stress-mediated cell death [38], and ADSCs have been found to have a protective role against retinal ischemic damage [85] and not only mediate angiogenesis via paracrine mechanisms in retinal endothelial cells but also promote retinal regeneration in vitro [86]. Tsuruma et al. identified progranulin as a major ADSC secreted with protective effects against retinal damage in vitro, and it may be a potential target in the context of degenerative retinal diseases [87].

Adipose mesenchymal stem cells (ASCs) and their pericyte-like differentiated phenotype (P-ASCs) may also have a beneficial effect on human retinal endothelial cells (HRECs) in high-glucose conditions. P-ASCs might be considered strong candidates for therapeutic approaches aimed at countering blood–retinal barrier (BRB) disruption in DR, as suggested by Lupo et al. [88]. The data presented herein provide evidence that ADSCs may serve as a promising therapeutic approach for DR [93]. Finally, hADSCs have also been induced to express the characteristics of endothelial cells (ECs) in vitro [94].

### 6.2. Exploration of ADSC-Mediated Immunomodulation

The mechanism of immunosuppression by ADSCs involves the cell-to-cell contact-mediated repression of function and maturation of T cells (CD4+ and CD8+ cells), B cells, dendritic cells (DCs), NK cells, neutrophils, and macrophages [95]. The secretion of immune-modulatory cytokines, such as nitric oxide (NO), indoleamine 2,3-dioxygenase (IDO), tumor necrosis factor-stimulated gene 6 (TSG6), prostaglandin E2 (PGE2), thrombospondin type 1 (TSP1), interleukins 6, 10 (IL6, IL10), TGFβ1, and HGF, has been shown to regulate these immune cells and anti-inflammatory responses by MSCs [96]. Moreover, MSC-derived exosomes are now known to modulate inflammation by promoting the polarization of macrophages from the pro-inflammatory M1 phenotype to the anti-inflammatory M2 phenotype, activation of regulatory T (Treg) cells, inhibition of B lymphocytes, and prevention of neutrophil mobilization [97,98].

Studies suggest that ADSCs have the potential to modulate immune responses in retinal diseases, such as uveitis and autoimmune retinopathies, by mitigating and suppressing retinal inflammation. As already stated, ADSCs also have immunomodulatory properties, important in the context of retinal disease treatment. This is achieved by the suppression of the activation and proliferation of T cells and the modulation of the function of other immune cells, including macrophages and dendritic cells. Finally, Yu et al. demonstrated that ADSCs secrete immunomodulatory factors (interleukin-10 (IL-10), transforming growth factor-beta (TGF-β), and indoleamine 2,3-dioxygenase (IDO)), important in the suppression of inflammatory responses [99].

## 7. Animal Studies

Animal studies are a mandatory step between in vitro investigations and human clinical trials to evaluate the safety and efficacy of ADSC-based therapies using animal models of retinal diseases. Animal models (rodents or non-human primates) mimic retinal diseases and enable researchers to study the therapeutic effects of ADSC transplantation, assess cell survival and integration, and investigate functional outcomes in the diseased retina. ADSCs can be delivered into the eyes of animal models through various routes, like intravitreal injection, subretinal injection, or transplantation, depending on the specific research objectives and the targeted retinal cell layer.

Intravitreal injections allow the ADSC paracrine effects on retinal cells to take effect; subretinal injections and transplantations place ADSCs in close proximity to the RPE and photoreceptor layers, facilitating direct interactions. Transplanted ADSCs can also be labeled with fluorescent dyes or genetic markers to track their “journey” within the eye and retina. The therapeutic effects of ADSCs within the retina can be evaluated through several means, including functional assessments (for example, with electrophysiological studies) or structural analysis, especially via optical coherence tomography (OCT), histological examination, and immunohistochemistry to detect cell markers and analyze gene expression (Table 2).

Transplanted ADSCs may differentiate into retinal cell types (RPE cells; PRs), thereby contributing to tissue regeneration. By investigating the fate and behavior of transplanted ADSCs, animal studies can explore the integration of transplanted ADSCs into the host retina and their ability to establish functional connections with existing retinal cells. Moreover, animal studies provide valuable insights for optimizing protocols, understanding the therapeutic effects, and identifying potential/ideal targets for ADSC-based therapies in retinal diseases [108,109,110].

Barzelay et al. showed that an ADSC-conditioned medium (ADSC-CM) exhibited a protective effect on the RPE layer and PRs from the damage [38], and in a study by Sugitani et al., conditioned media from hADSCs inhibited retinal damage in vitro and in vivo, showing that hADSC-CM has neuroprotective effects against light-induced retinal damage, thus suggesting that hADSCs may have therapeutic potential in retinal degenerative diseases via their secreted proteins, without requiring transplantation [100]. Tsuruma et al. also evaluated the protective effect of ADSCs and ADSC-CM against retinal damage. ADSCs were injected intravitreally in a mouse model of light-induced retinal damage and were shown not only to induce the recovery of retinal function, as measured via electroretinogram, but also to inhibit outer nuclear layer thinning without engraftment; these findings suggest that ADSC-CM and progranulin secreted by this class of cells have neuroprotective effects in the light-induced retinal damage model [87].

In Kadkhodaeian et al.’s work on albino Sprague-Dawley rats, which used a sodium iodate model for the RPE injury, ADSCs were found to survive for 4 weeks after transplantation and to migrate into the RPE layer in the injured retina [101]. More recently, Dov et al. examined the transplantation of ADSCs in a co-culture system with organotypic ischemic retinas, and RGC recovery was demonstrated. Since there was no advantage from the direct contact of ADSCs with RGCs, the beneficial effect seen is likely to be related to the paracrine activity of ADSCs, and the data correlated well with the secretion profile of ADSCs’ anti-apoptotic and pro-proliferative cytokines [37].

Zhou et al. explored injecting hADSCs intravitreally in mice with oxygen-induced retinopathy (OIR) to check the cell movement, fusion, proliferation, and life span in vivo. After induction, hADSCs expressed von Willebrand Factor (vWF), the cell marker of endothelial cells, but they were distributed above the retina and did not fuse with it, gathering in the central and peripheral areas, which is the lesion area of the OIR model. Five days after hADSC intravitreal administration, the area of neovascularization was reduced by 94.83% compared with the OIR control group that did not receive hADSCs. Hematologic staining and electron microscopy did not show noticeable proliferation and degeneration of the retina in the “treatment group”, highlighting the potential application of intraocular hADSCs [94].

Mendel et al. found that the intravitreal injection of ASCs in an oxygen-induced retinopathy mouse model and Akimba diabetic mice models resulted in the integration of the injected cells in the retinal microvessels and exhibited pericyte-like functions, with the normalization of the retinal microvasculature and prevention of capillary loss [78].

MSCs have been tested for pericyte replacement in several other animal models, showing the repair and regeneration of DR-damaged vasculature. Fiori et al. showed that ADSCs may be potentially therapeutically active in DR by restoring angiogenic deficits in retinal endothelial cells via the secretion of proangiogenic factors. However, the authors also advocate thorough risk assessment regarding the timing of ADSC-based cell therapy, which may be advantageous in the early stage of DR but possibly detrimental in later neo-angiogenic stages of DR [111].

Safwat et al. also reported that intraocular and subconjunctival administration of adipose stem-cell-derived exosomes ameliorated the characteristic retinal degeneration in streptozotocin-induced diabetic rabbits [39], whereas Ezquer et al. showed that the intravitreal administration of ADSCs triggered an effective cytoprotective microenvironment in the retina of diabetic mice and resulted in a significant increase in intraocular levels of NGF, FGF2, and GDNF, preventing RGC loss and reducing oxidative stress in the retina. Furthermore, the injected cells also differentiated into RGCs, astrocytes, and pericytes in vivo, and, therefore, ADSCs may represent an interesting tool in order to prevent DR [76].

In another study by Rajashekar et al., a strategy to regenerate the retinal vasculature and neuronal cell integrity via intravitreal injection of ADSCs in streptozotocin-induced murine models of early DR was proposed. ADSCs were reported to integrate into retinal perivasculature, with consequent reconstitution of the blood–retinal barrier within several weeks, suggesting a pericyte-like function of the injected cells [77].

Moreover, Elshaer et al. hypothesized that hADSCs positive for the pericyte marker CD140b therapeutically rescue early-stage DR features in Ins2Akita mice by mitigating the retinal complications of diabetes, either directly or indirectly via their secreted paracrine factors [79]. Cronk et al. examined how injected murine ADSCs affect the retina microvasculature in a mouse model of DR. The results showed that ADSCs obtained from healthy mice secrete angiogenic growth factors and promote retinal vascular stability when they are injected intravitreally. The authors’ work also suggests that ADSCs obtained from diabetic mice have a diminished ability to support the retinal vasculature in this mouse model of retinal vasculopathy [80].

Gounari et al. evaluated the use of a stem-cell-based therapy for retinal vein occlusion (RVO) in a pharmaceutically induced animal model of RVO; the study investigated the effect of the paracrine action of ADSCs combined with anti-VEGF nanocarriers, which allowed for a sustained release of anti-VEGF, resulting in a progressive reduction in neo-vascularization [102].

The subretinal transplantation of hADSCs from orbital fat (OADSCs) in a mouse model of retinal degeneration led to the restoration of the RPE layerm whilst the transplantation of hADSCs from abdominal subcutaneous fat (ABASCs) resulted in significant restoration of the photoreceptor layer. Taken together, a lineage-specific therapeutic effect for either OASCs or ABASCs in retinal regeneration was demonstrated [103].

Xuqian et al. investigated whether hADSC transplantation would improve the healing process of a rabbit model of retinal holes by creating retinal holes in the left eyes of 20 New Zealand white rabbits and randomly filling them with hADSCs (transplantation group) or phosphate-buffered saline (control group). Using frequency-domain optical coherence tomography (OCT) and immunofluorescence, it was clear that transplanted hADSCs could engraft in the retinal hole of the model, accelerating the healing process and promoting injury recovery [104].

Yu et al. reported that adipose-derived MSC exosomes ameliorated laser-induced retinal injury in mice and prevented extensive photoreceptor cell damage via the downregulation of monocyte chemotactic protein-1 [105]. However, although stem-cell-derived secretome-based therapies for the treatment of retinal degenerative diseases show promise, their exact mechanism of action and the possible cellular targets are unclear [112]. Previously, Jha et al. demonstrated that one single intravitreal injection of an hADSC-concentrated conditioned medium (hADSC-CCM) protected against the visual deficits of mild traumatic brain injury (mTBI) in adult C57Bl/6 mice [106], and the loss of visual acuity and contrast sensitivity was mitigated 5 weeks post-blast injury.

Interestingly, blast mice showed increased retinal glial fibrillary acidic protein (GFAP) immunoreactivity in Müller cells, indicative of an astrocytic response to the retinal injury, whilst blast mice that received hADSC-CCM did not show this Müller cell gliosis, and, therefore, a novel neuroprotective role for hADSC-CCM in the rescue of the visual deficits and pathologies of mTBI via the restoration of Müller cell health has been suggested [107].

Finally, the subretinal transplantation of hADSCs in Royal College of Surgeons (RCS) rats effectively delayed retinal degeneration, enhanced retinal cell survival, and improved visual function. This was thought to be mainly linked to hADSC-dependent anti-apoptotic and neuroprotective effects through the secretion of growth and neurotrophic factors including VEGF. It was concluded that the clinical application of hADSCs definitely merits further investigation and interest [109].

## 8. Human Studies

Current ADSC-based human studies in retinal disease are primarily focused on safety and preliminary efficacy assessments and, therefore, typically involve a limited number of patients. Patients with severe retinal diseases, such as advanced AMD or retinitis pigmentosa (RP), are monitored for adverse events, functional changes (visual acuity, visual field, contrast sensitivity, electrophysiology), and structural improvements (i.e., retinal thickness and morphology on OCT) following transplantation of autologous or allogeneic ADSCs. Human clinical trials are crucial for evaluating the safety, feasibility, and efficacy of ADSCs in retinal disease, in order to assess safety, visual outcomes, and functional improvements.

### 8.1. Clinical Trials Investigating ADSC Transplantation in Retinal Disease

ADSCs can be delivered to the human retina via intravitreal injection, subretinal injection, or transplantation with a carrier scaffold, and their therapeutic potential has been tested in various retinal diseases, including AMD, RP, and DR [113]. The trials aim to determine the safety and feasibility of ADSC transplantation, evaluate its therapeutic effects, and establish optimal transplantation protocols. They often involve careful patient selection and follow-up examinations to monitor the progression of the disease and treatment outcomes.

Several trials on ADSC administration for the treatment of retinal degenerative diseases exist; most of them are in phase 1 or 2 and are focused on safety. These cell applications were studied on patients suffering from various retinal diseases, ranging from retinitis pigmentosa and other rare inherited retinal disorders to more common retinopathies, such as DR, retinal vasculopathies, and macular pathologies of medical and surgical interest, such as AMD, RP and macular holes [114,115,116]. However, in most of these trials, stem cells are derived from other tissue than adipose tissue, mostly autologous bone marrow, but also the umbilical cord including Warton’s jelly [33].

Only one study applied cells obtained from adipose tissue. Oner et al. injected ADSCs under the retina of 11 patients affected by RP and followed them for 6 months. The experimental procedure consisted of complete vitrectomy, subretinal injection of cells, air tamponade, and face-down posturing for 1 day after surgery to spread the cells in the subretinal space. Significant improvements in BCVA (from 20/2000 to 20/200), perimetry, and ERG were observed in one patient. Slight improvements in BCVA and brighter color vision were reported by three patients, whereas the remaining seven patients showed no BCVA changes, with five of them having light perception at baseline. Choroidal neovascularization (CNV) at the implantation site occurred after surgery in one case and required intravitreal treatment with anti-VEGFs. Five patients developed an epiretinal membrane, which was treated with vitrectomy revision, peeling, and silicon oil tamponade [117]. 

### 8.2. Safety and Feasibility Assessments

Safety assessments involve monitoring for adverse events, such as infections, inflammation, or immune reactions, associated with the transplantation procedure or the presence of transplanted cells. Feasibility assessments evaluate the practicality and effectiveness of ADSC transplantation in the clinical setting. Factors, such as cell viability, survival, and integration within the host retina, as well as the surgical technique and delivery method, are evaluated to determine the feasibility of ADSC transplantation as a viable treatment option. Limoli et al. observed favorable outcomes among elderly patients with non-exudative AMD after undergoing the suprachoroidal grafting of mature adipocytes and ADSCs in the stromal vascular fraction (SVF), which was enriched with platelet-rich plasma (PRP). The study showed no complications and an improvement in scotopic electroretinographic scores in these patients [118]. Additionally, this group documented objective measurements after performing the autologous transplantation of adipocytes, ADSCs in the SVF, and platelet-rich plasma (PRP) in the suprachoroidal space of patients diagnosed with dry AMD [119].

### 8.3. Visual Outcomes and Functional Improvements

Human studies also aim to assess visual outcomes and functional/structural improvements following ADSC transplantation. Functional electrophysiological assessments provide objective data on the improvement in or preservation of retinal function following ADSC transplantation. Visual acuity measurements and visual-field testing can be utilized to evaluate subjective measures, whilst imaging techniques such as OCT are used to evaluate structural changes in the retina. Furthermore, quality-of-life assessments and patient-reported outcomes are considered to evaluate the overall impact of ADSC transplantation on visual function, daily activities, and well-being. It is important to note that human studies in the field of ADSC transplantation in retinal disease are still in the early stages, and more research is needed to establish long-term safety, efficacy, and optimal protocols for treatment [114,120].

## 9. Conclusions and Future Directions

This comprehensive review highlighted the use of ADSCs as a promising therapeutic approach for retinal diseases. ADSCs possess unique properties, including their easy accessibility, abundance, and ability to differentiate into various cell types, making them an attractive candidate for regenerative medicine in the field of ophthalmology [33,121].

This review provided a brief overview of the roles of SCs in ophthalmology. SCs, particularly ADSCs, were introduced as a potential solution due to their regenerative and immunomodulatory properties. This review discussed ADSCs’ isolation and characterization techniques, highlighting their advantages, such as their abundance in adipose tissue and minimal invasiveness during extraction. The differentiation potential of ADSCs into retinal cell types and their immuno-modulatory properties were also examined. Furthermore, this review explored in vitro, animal, and human studies involving ADSCs in the context of retinal disease. The findings demonstrated the potential of ADSCs to differentiate into retinal cells, promote retinal cell survival, and modulate inflammatory responses. Animal studies provided evidence of functional and structural improvements in the retina following ADSC transplantation. Moreover, preliminary findings from human studies indicated the safety and feasibility of ADSC transplantation in retinal diseases, along with some promising visual and functional outcomes.

The therapeutic effects of ADSCs in retinal diseases were discussed, focusing on their ability to modulate retinal inflammation and immune responses, promote cell survival and regeneration, enhance vascularization, and provide neuroprotection. However, it is important to acknowledge the challenges and limitations associated with ADSC-based therapies, including the heterogeneity of ADSC populations, standardization of protocols, and the need for improved cell survival and integration within the host tissue. ADSC populations can vary in terms of their differentiation potential and therapeutic properties; this heterogeneity makes it challenging to ensure therapeutic outcomes. Moreover, the characteristics of ADSCs can vary depending on the donor’s age, health conditions, and lifestyle. This variability poses challenges in standardizing the treatment and predicting outcomes. The potential risk linked to the secretion of vascular growth factors can lead to complications, like pathological neovascularization and retinal fibrosis, contributing to the progression of certain retinal diseases, like DR. The clinical use in humans is restricted to one trial with 11 subjects, of which 5 developed an epiretinal membrane and required vitrectomy revision and 1 developed choroidal neovascularization at the implantation site. The correct timing and dosage of ADSCs are crucial; a precise balance must be obtained in order to ensure that the secreted growth factors promote proper, controlled angiogenesis without causing the previously mentioned side effects.

Despite these challenges, this review reinforced the potential of ADSCs as a promising therapeutic approach for retinal diseases. The regenerative and immunomodulatory properties of this class of SCs offer a multi-faceted approach to address the complex pathophysiology of retinal diseases. Combining ADSC-based therapies with other treatment modalities, such as anti-VEGF therapy or gene therapy, may further enhance the treatment outcomes. However, further research and well-designed clinical trials are necessary to establish the long-term efficacy, safety, and optimal protocols for ADSC transplantation in retinal diseases. Large-scale randomized controlled trials with standardized outcome measures are needed to validate the promising results observed in preclinical and early clinical studies.

In conclusion, ADSCs hold great potential for the treatment of retinal diseases. Continued research, collaboration between scientists and clinicians, and regulatory support are essential to advance the field and translate ADSC-based therapies into routine clinical practice. With further advancements and refinements, ADSC-based treatments may revolutionize the management of retinal diseases, providing new hope for patients with vision impairment and improving their quality of life. 

## Figures and Tables

**Table 1:** Relevant in vitro studies investigating the effects of ADSCs on retinal diseases.

ADSC Origin	Retinal Disease	Effect on Retinal Diseases	References
decellularized matrix manufactured using 0.5% ammonium hydroxide Triton + 20 mmol/L NH4OH in combination with DNase solution	Not specified	− enhancing the proliferation of retinal progenitor cells (RPCs) by activating Akt and Erk phosphorylation − promotion of the differentiation of RPCs toward retinal neurons, especially rhodopsin- and recoverin-positive photoreceptors	Ji et al. [82]
Human-ADSCs (hASCSs) induced by RPE-conditioned medium (RPECM)	age-related macular degeneration (AMD)	− protein levels of retinoid isomerohydrolase (RPE65), cytokeratin (CK8) and Bestrophin were significantly increased in RPECM-treated hADSCs − RPECM induces hADSCs to differentiate into RPE cells with higher proliferative and migratory potentials	Zhang et al. [46]
Human adipose tissue, the coding region of the human PAX6 (5a) gene isoform was cloned and lentiviral particles were propagated in HEK293T	Not specified	− polymerase chain reaction and immunocytochemistry confirmed the differentiation of some neural retinal cells and RPE cells. Thus, PAX6 (5a) transcription factor expression, together with medium supplemented with fibronectin, is able to induce the differentiation of hADSCs into retinal progenitors, RPE cells and photoreceptors	Razanejad et al. [49]
human orbital ADSCs generated by enzyme (EN) and explant (EX) culture methods	Not specified	− orbital ADSCs isolated by direct explants culture show earlier and stronger expressions of markers towards retinal photoreceptor differentiation than those generated by conventional EN method	Xu et al. [83]
adipose tissue, ADSCs cultured in a conditioned medium (CM) from an RPE cell line (ARPE-19 CM)	Not specified	− the presence of growth factors produced by ARPE-19 cells in tissue culture induces ADSCs to express neural differentiation markers typical of the neuronal and glial cells of the retina	Mannino et al. [84]
adipose tissue	Not specified	− adding specific growth factors like retinoic acid, fibroblast growth factor (FGF), or Wnt signaling activators, can promote the differentiation of ADSCs into RPE-like cells, which exhibit characteristic RPE morphology, express RPE-specific markers (e.g., RPE65, Bestrophin-1), and display functional RPE properties	Shen et al. [45]
Human ADSCs	Hypoxic ex vivo organotypic cultures of retina	− transplantation of ADSCs in a co-culture system with organotypic ischemic retinas resulted in retinal ganglion cells (RGCs) recovery.	Dov et al. [37]
ADSCs	Not specified	− ADSCs exhibited enhanced migration when exposed to conditioned medium of oxidative stressed RPE cells obtained by hydrogen peroxide − ADSCs showed the potential to migrate towards damaged RPE cells and protect them in a paracrine manner from cell death induced by oxidative stress	Barzelay et al. [38]
Induced MSCs (iMSCs) generated by differentiating the induced pluripotent stem cells (iPSC)	Not specified	− iMSC can enhance differentiation of T cells into transcription factor forkhead box protein P3 T regulatory cells (Foxp3 Tregs) in vitro and therapeutically improve the retina’s immune function	Agrawal et al. [85]
ADSCs	Not specified	− demonstration of substantial role for CD140b [platelet-derived growth factor receptor- β (PDGFR-β)] in the intrinsic abilities of ASCs and their angiogenic influence on human retinal endothelial cells (HREs)	Periasamy et al. [86]
mouse subcutaneous tissue	light-induced retinal damage	− H_2_O_2_- and light-induced cell death was reduced in a photoreceptor cell line with ASC-CM but not with mature adipocyte-conditioned medium	Tsuruma et al. [87]
pericyte-like differentiated phenotype of ADSCs (P-ASCs)	Diabetic retinopathy (human retinal endothelial cells (HRECs) in high glucose conditions [25 mM glucose, HG])	− P-ASCs might be considered valuable candidates for therapeutic approaches aimed at countering blood-retinal barrier (BRB) disruption in DR	Lupo et al. [88]

**Table 2:** Experimental animal models of ADSC-based therapy for retinal disorders.

Induction of Retinal Diseases	Species	Treatment	Results	Reference
sodium iodate model with consequent oxidative stress, acute injury to RPE, and progressive ongoing retinal damage	Wild-type C57BL mice	subretinal transplantation of hADSCs	ADSCs are able to home in on damaged RPE cells and protect against damage to the RPE and PR layers caused by oxidative stress	Barzelay et al. [38]
light-induced retinal photoreceptor damage (under controlled lighting conditions [12/12 h light/dark cycle])	Male adult ddY albino mice	Not available	− hADSC-Conditioned Medium (hADSC- CM) inhibited photoreceptor degeneration and retinal dysfunction after exposure to light − proteins secreted by hASCs, such as the tissue inhibitor of metalloproteinase-1 (TIMP-1) and the secreted protein acidic and rich in cysteine (SPARCC), protected against light-induced damage	Sugitani et al. [100]
light-induced retinal photoreceptor damage (under controlled lighting conditions [12/12 h light/dark cycle])	Male adult ddY mice and C57BL/6-Tg (CAG-EGFP) mice	Murine ADSCs intravitreally injected	ADSC-CM significantly inhibited photoreceptor degeneration and retinal dysfunction after light exposure. Progranulin was identified as a major secreted protein of ASCs that showed protective effects against retinal damage	Tsuruma et al. [87]
Retinal pigmented epithelium damage induced by retro-orbital sinus sodium iodate injection	Sprague-Dawley albino rats	Murine ADSCs transplanted in the subretinal space	ADSCs survived for 4 weeks after transplantation and migrated into the RPE layer	Kadkhodaeian et al. [101]
oxygen-induced retinopathy (OIR)	Pregnant C57BL/6J mice	hADSCs intravitreally injected	five days after the hADSC intravitreal injection, the area of neovascularization was reduced by 94.83% compared with that of the OIR group	Zhou et al. [94]
oxygen-induced retinopathy (OIR)	NOD SCID mice (Charles River)	hADSCs intravitreally injected	ADSC-derived pericytes can integrate with retinal vasculature, adopting both pericyte morphology and marker expression,	Mendel et al. [78]
Diabetes Mellitus induced by intravenous injections of streptozotocin (STZ)	Male rabbits	Rabbit ADSCs-exosomes	potency of rabbit ADSCs-derived exosomes in retinal repair	Safwat et al. [39]
Diabetes Mellitus induced by intraperitoneal injections of streptozotocin (STZ)	C57BL/6 and C57BL76-Tg(ACTB-EGFP)1Obs mice	Murine ADSCs intravitreally injected	increased the intraocular levels of several potent neurotrophic factors (nerve growth factor, basic fibroblast growth factor and glial cell line-derived neurotrophic factor) and reduced the oxidative damage in the diabetic retina	Ezquer et al. [76]
Diabetes Mellitus induced by intraperitoneal injections of streptozotocin (STZ)	Athymic nude rats (Hsd:RH-Foxn1^rnu^)	hADSCs intravitreally injected	ADSCs are able to rescue the neural retina from hyperglycemia-induced degeneration, resulting in importantly improved visual function	Rajashekhar et al. [77]
early-stage Diabetic Retinopathy mouse model	Ins2^Akita^ heterozygote male mice and age-matched C57BL/6J (WT) male mice	hADSCs intravitreally injected	ADSCs or their secreted factors mitigate retinal complications of diabetes	Elsaher et al. [79]
Diabetic Retinopathy mouse model	Akimba mouse	mouse ADSCs (mADSCs)/media conditioned by mASCs intravitreally injected	ADSCs taken from diabetic sources have an impaired ability to stabilize the microvasculature in diabetic retinopathy	Cronk et al. [80]
intravitreal injections of MEK kinase inhibitor induced animal model of Retinal Vein Occlusion (RVO)	New Zealand rabbits	intravitreal administration of rabbit ADSCs combined with encapsulated anti-VEGF in thiolated chitosan nanocarriers (nanoThioCHI)	a stem cell-based therapy for RVO is proposed, accompanied by sustained release of anti-VEGF, in order to combine the paracrine action of ASCs and the progressive reduction of neovascularization.	Gounari et al. [102]
mouse model of retinal degeneration	sodium iodate mice model	Subretinal transplantation of human orbital (OADSCs) and abdominal ADSCs (ABASCs)	subretinal transplantation of OADSCs in a mouse model of retinal degeneration led to restoration of the RPE layer while transplantation of ABASCs resulted in a significant restoration of the photoreceptor layer	Krief et al. [103]
Rabbit Model of Experimental Retinal Holes	New Zealand rabbits	hADSCs intravitreally injected	Transplanted hAD-MSCs could engraft in the retinal hole of a rabbit model, and clearly accelerated the healing process and ameliorated injury recovery	Xuqian et al. [104]
Krypton laser-induced retinal injury	C57BL/6 mice	Mouse ADSC-derived exosomes (MSC-Exos) intravitreally injected	MSC-Exos ameliorate laser-induced retinal injury partially through down-regulation of monocyte chemotactic protein MCP-1	Yu et al. [105]
mouse model of visual deficits following mild traumatic brain injury (mTBI)	C57BL/6 mice	ADSC concentrated conditioned medium (ADSC-CCM) from cells pre-stimulated with inflammatory cytokines (ASC-CCM) intravitreally injected	Injection of ADSC-CCM mitigates loss of visual acuity and contrast sensitivity four weeks post blast injury.	Jha et al. [106]
mouse model of visual deficits following mild traumatic brain injury (mTBI)	C57Bl/6 mice	Human ADSC concentrated conditioned medium (ADSC-CCM) intravitreally injected	novel neuroprotective role for ASC-CCM in the rescue of the visual deficits and pathologies of mTBI via restoration of Müller cell health	Jha et al. [107]

## Data Availability

Not applicable.
